# Temporal changes in the risk of six-month post-COVID symptoms: a national population-based cohort study

**DOI:** 10.1093/aje/kwae174

**Published:** 2024-07-03

**Authors:** Anne Pastorello, Laurence Meyer, Joël Coste, Camille Davisse-Paturet, Xavier de Lamballerie, Maria Melchior, Sophie Novelli, Delphine Rahib, Nathalie Bajos, Cécile Vuillermoz, Jeanna-Eve Franck, Carmelite Manto, Alexandra Rouquette, Josiane Warszawski, EpiCov study group

**Affiliations:** Paris-Saclay University, University of Versailles Saint-Quentin-en-Yvelines, National Institute of Health and Medical Research, Center for Epidemiology and Population Health, Le Kremlin-Bicêtre, France; Paris-Saclay University, University of Versailles Saint-Quentin-en-Yvelines, National Institute of Health and Medical Research, Center for Epidemiology and Population Health, Le Kremlin-Bicêtre, France; Epidemiology and Public Health Department, Assistance Publique-Hôpitaux de Paris Université Paris-Saclay, Le Kremlin-Bicêtre, France; Department of Non-Communicable Diseases and Injuries, French Public Health Agency, Saint-Maurice, France; Paris-Saclay University, University of Versailles Saint-Quentin-en-Yvelines, National Institute of Health and Medical Research, Center for Epidemiology and Population Health, Le Kremlin-Bicêtre, France; Emerging Viruses Unit, Aix-Marseille University, French National Research Institute for Sustainable Development 190 - National Institute of Health and Medical Research, University Hospital Institute Méditerranée Infection, Marseille, France; Pierre Louis Institute of Epidemiology and Public Health, Sorbonne University, National Institute of Health and Medical Research, Paris, France; Paris-Saclay University, University of Versailles Saint-Quentin-en-Yvelines, National Institute of Health and Medical Research, Center for Epidemiology and Population Health, Le Kremlin-Bicêtre, France; Department of Non-Communicable Diseases and Injuries, French Public Health Agency, Saint-Maurice, France; Interdisciplinary Institute of Social Issues – social sciences, politics, health, French National Center for Scientific Research, School for Advanced Studies in Social Sciences, National Institute of Health and Medical Research, Aubervilliers, France; Paris-Saclay University, University of Versailles Saint-Quentin-en-Yvelines, National Institute of Health and Medical Research, Center for Epidemiology and Population Health, Le Kremlin-Bicêtre, France; Interdisciplinary Institute of Social Issues – social sciences, politics, health, French National Center for Scientific Research, School for Advanced Studies in Social Sciences, National Institute of Health and Medical Research, Aubervilliers, France; Paris-Saclay University, University of Versailles Saint-Quentin-en-Yvelines, National Institute of Health and Medical Research, Center for Epidemiology and Population Health, Le Kremlin-Bicêtre, France; Paris-Saclay University, University of Versailles Saint-Quentin-en-Yvelines, National Institute of Health and Medical Research, Center for Epidemiology and Population Health, Le Kremlin-Bicêtre, France; Epidemiology and Public Health Department, Assistance Publique-Hôpitaux de Paris Université Paris-Saclay, Le Kremlin-Bicêtre, France; Paris-Saclay University, University of Versailles Saint-Quentin-en-Yvelines, National Institute of Health and Medical Research, Center for Epidemiology and Population Health, Le Kremlin-Bicêtre, France; Epidemiology and Public Health Department, Assistance Publique-Hôpitaux de Paris Université Paris-Saclay, Le Kremlin-Bicêtre, France

**Keywords:** long COVID, post-COVID symptoms, SARS-Cov-2, COVID-19, cohort, population-based, survey weights, multiple imputation

## Abstract

It is unclear how the risk of post-COVID symptoms evolved during the pandemic, especially before the spread of Severe Acute Respiratory Syndrome Coronavirus 2 variants and the availability of vaccines. We used modified Poisson regressions to compare the risk of six-month post-COVID symptoms and their associated risk factors according to the period of first acute COVID: during the French first (March-May 2020) or second (September-November 2020) wave. Nonresponse weights and multiple imputation were used to handle missing data. Among participants aged 15 years or older in a national population-based cohort, the risk of post-COVID symptoms was 14.6% (95% confidence interval [CI], 13.9%-15.3%) in March-May 2020, vs 7.0% (95% CI, 6.3%-7.7%) in September-November 2020 (adjusted relative risk [RR], 1.36; 95% CI, 1.20-1.55). For both periods, the risk was higher in the presence of baseline physical condition(s), and it increased with the number of acute symptoms. During the first wave, the risk was also higher for women, in the presence of baseline mental condition(s), and it varied with educational level. In France in 2020, the risk of six-month post-COVID symptoms was higher during the first than the second wave. This difference was observed before the spread of variants and the availability of vaccines.

## Introduction

After an acute Severe Acute Respiratory Syndrome Coronavirus 2 (SARS-Cov-2) infection, some subjects develop persisting symptoms, lasting sometimes for months or years,^1^^,^^2^ that significantly impact their daily activities.^3^^,^^4^ Although the causes of these symptoms remain largely unknown, there is evidence that SARS-Cov-2 can damage many organ systems.^5^^,^^6^ Several mechanisms, including viral persistence of SARS-Cov-2, immunity dysregulation, clotting/endothelial abnormality, dysfunctions in neurological signaling, or postintensive care syndrome for severe cases, could explain post-COVID symptoms.^7^^,^^8^

The risk of post-COVID symptoms after SARS-Cov-2 infection is estimated in the literature to range from 4% to 80%.^7^^,^^9^^‑^^11^ The reasons for this wide range could be the use of different definitions of post-COVID symptoms (type, onset, and duration),^12^^,^^13^ the heterogeneity of the populations under study, or the frequent use of convenience samples.^10^^,^^14^ Concerning risk factors, findings also vary across studies. Most studies found a higher risk of post-COVID symptoms for women, older subjects, severe COVID cases, and subjects with preexisting mental or physical comorbidities.^7^^,^^15^^,^^16^ However, some socioeconomic or environmental factors, such as deprivation,^17^ isolation,^18^ or air pollution exposure,^19^ were also recently documented as risk factors for post-COVID symptoms. In addition, there is inconsistent evidence on the role of ethnicity^6^^,^^7^^,^^17^^,^^20^ in the risk of post-COVID symptoms.

Differences in temporal settings between the studies could also explain this lack of consistent evidence. After 2020, the risk of post-COVID symptoms may have decreased with the availability of anti-SARS-Cov-2 vaccines,^7^^,^^21^^,^^22^ and evolved with the appearance of new SARS-Cov-2 variants.^23^^‑^^25^ This risk also probably varied according to the time of the subject’s initial acute COVID episode, even before the spread of variants or the availability of anti-SARS-Cov-2 vaccines. Since the beginning of the COVID-19 pandemic, protective measures against SARS-Cov-2 and healthcare for infected subjects have greatly improved. In the first semester of 2020, very little was known on the aftermaths of SARS-Cov-2 infection. At that time, infected subjects may have been all the more affected by anxiety, potentially increasing the risk of post-COVID mental and physical symptoms.^18^^,^^26^ In addition, the stringent lockdowns that were applied in most countries during this period^27^ may have delayed recovery for infected people by reducing their access to healthcare or their ability to engage in behaviors that promote physical and mental health.^28^^‑^^30^ These two phenomena probably reduced thereafter, with the improvement of knowledge on COVID-19 and the efficacy of alternative preventive measures, such as mandatory face covering in public spaces or wide scale screening for SARS-Cov-2, at least in high or medium income countries.^28^ Also, the risk of physical or psychological sequelae after a severe acute infection may have declined with the documented improvement of acute healthcare in the course of the pandemic, especially in intensive care units.^31^^,^^32^

Evidence of temporal changes in the risk of post-COVID symptoms could therefore bring insight into the potential underlying mechanisms of these symptoms, as it would suggest that their persistence may depend on the context of the acute COVID episode. These changes could also partly explain heterogeneous estimates of post-COVID symptoms risk across studies conducted in various temporal settings. A French study conducted in August 2021 found that post-COVID symptoms were associated with a history of SARS-Cov-2 infection during the first epidemic wave (odds ratio 1.82; 95% confidence interval [CI], 1.55-2.15).^33^ However, this study was based on a convenience sample recruited through social media and did not take into account sociodemographic or health-related characteristics that could explain the higher risk of post-COVID symptoms in subjects who were exposed to SARS-Cov-2 during the first wave in France.^34^

### Objectives

Our study used data from the French national random population-based prospective cohort Epidémiologie et conditions de vie (EpiCov) conducted in subjects aged 15 or older^34^^‑^^36^ to explore whether the risk of post-COVID symptoms varied in 2020, before the arrival of the alpha variant^37^ or the availability of anti-SARS-Cov-2 vaccines.^38^ We compared the risk of post-COVID symptoms at six months or more, adjusted for potential sociodemographic or health-related confounders, according to the period of the first acute COVID episode: during the French first (March-May 2020) or second (September-November 2020) epidemic wave. We expected a higher risk for subjects infected during the first wave, which was marked by uncertainty regarding SARS-Cov-2 infection aftermaths and healthcare, and by the most stringent lockdown in France.^27^ We also explored sociodemographic and health-related risk factors for post-COVID symptoms separately for the two periods in stratified analyses and tested the effect modification of the period on these risk factors.

## Methods

### The EpiCov cohort: Design and data collection

**Figure 1:**
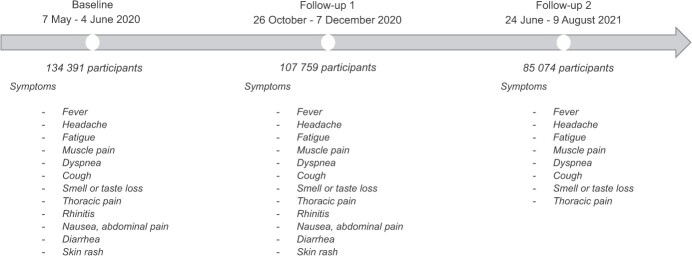
EpiCov French national cohort (2020-2021) study timeline and data collection on COVID symptoms at baseline and two follow-ups.

The socio-epidemiologic EpiCov cohort was set up in May 2020 to study the spread of SARS-Cov-2 infection in general population and its relationship with health and living conditions. Individuals aged 15 years or older living in France were randomly selected from the national Fichiers démographiques sur les logements et les individus (FIDELI) tax and administrative database, which covers more than 96% of the population. The probability sampling design and data collection have been described elsewhere.^34^^‑^^36^ Briefly, data were collected through computer-assisted web interviews or computer-assisted telephone interviews. Of the 371 000 subjects eligible for interview, 134 391 (36.2%) participated at baseline, and respectively 107 759 (29.0%) and 85 074 (22.9%) at the first and second follow-ups (Figure 1). Weights derived from nonresponse models and calibration on margins from the census were used to take the EpiCov design and nonparticipating bias into account at baseline and at each follow-up^35^ (Figure 1).

The questionnaires included, in particular, information on the time of the first occurrence, duration (one week, four weeks, or more), and current presence of sudden and unusual symptoms among the following: fever, headache, fatigue, muscle pain, cough, dyspnea, smell or taste loss, and thoracic pain. At baseline and first follow-up, this list also included nausea, diarrhea, rhinitis, and skin rash (Figure 1).

### Study population

In the current analysis, we included subjects living in mainland France, who participated at baseline (May 2020) as well as first (November 2020) and second (July 2021) follow-ups, if they had a probable symptomatic SARS-CoV2 infection, hereafter referred to as “acute COVID,” occurring for the first time either during the first (March-May 2020) or second epidemic wave (September-November 2020). We defined acute COVID, according to the European Center for Disease Prevention and Control (ECDC), as a sudden and unusual onset of taste/smell loss, fever, cough, dyspnea, or thoracic pain.^39^ Participants who had an acute COVID before the first wave, or between the two waves, or who reported more than one acute COVID episode at the time of the first follow-up, were excluded.

### Main outcome: Six-month post-COVID symptoms

**Figure 2:**
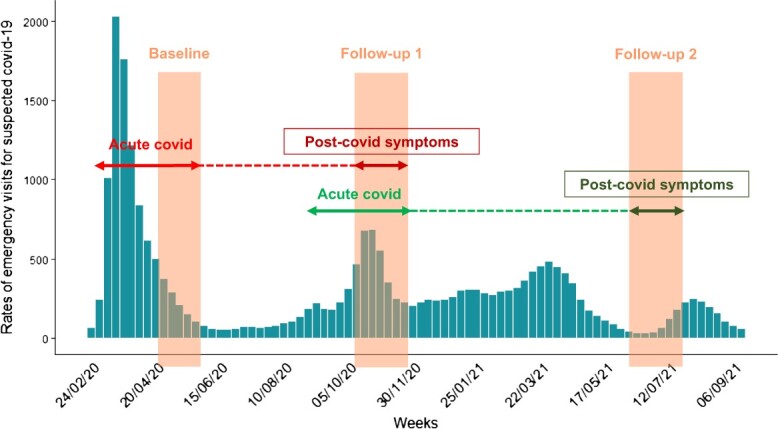
Data collection period on acute COVID and post-COVID symptoms in the Epicov French national cohort, in relation to the evolution of the pandemic in France (2020-2021). The rates of emergency visits (axis y) per week (axis x) for suspected COVID-19 were extracted from the public Géodes website of the French Public Health Agency (https://geodes.santepubliquefrance.fr), which collects routine data from emergency facilities. These rates, calculated for 10 000 visits, represent the weekly proportion of emergency visits with a medical diagnosis of suspected COVID-19 (ICD-10 codes: U07.1, U07.10, U07.11, U07.12, U07.14, U07.15, U04.9, B34.2, B97.2), among all emergency visits with a recorded medical diagnosis.

Six-month post-COVID symptoms included fever, headache, fatigue, muscle pain, cough, dyspnea, taste/smell loss, or thoracic pain, present during the acute COVID episode and persistent at six months or more. We considered a symptom as persistent at six months or more if it was ongoing at the time of the first follow-up (November 2020) for participants with an acute COVID during the first epidemic wave (mid-March-May 2020), or ongoing at the time of the second follow-up (July 2021) for participants with an acute COVID during the second epidemic wave (mid-September-November 2020, Figure 2). Participants who did not answer any question on post-COVID symptoms, or who reported post-COVID symptoms with no onset date or duration, had missing data on post-COVID symptoms (Figure 2).

### Main exposure: Period of the first acute COVID episode

The main exposure was the period of the first acute COVID episode: from mid-March to May 2020 (first epidemic wave), or from mid-September to November 2020 (second epidemic wave). Participants who did not answer any question on acute symptoms, or who reported acute symptoms with no onset date, had missing data on the period of acute COVID.

### Covariates

Sociodemographic baseline characteristics included gender, age, deprivation status obtained from the 2018 tax declaration in the FIDELI database (above or below the poverty threshold, ie, 60% of the median national annual income), educational level, migratory status (majority population, first-generation European immigrants, second-generation European immigrants, first-generation non-European immigrants, second-generation non-European immigrants),^34^ and occupational status (frontline healthcare workers, other frontline workers, nonfrontline workers, and nonworkers).^40^ We also included potential indicators of stressful living conditions during the pandemic^41^^,^^42^: the age of the youngest child in the household and any history of SARS-Cov-2 symptoms (cough or fever) among members of the household.

Self-reported health-related characteristics included the severity of acute COVID, approached by the number of acute symptoms and by COVID-related hospitalization. They also included body mass index (BMI; ≤25 kg/m^2^, >25 kg/m^2^), baseline presence or absence of any physical or mental chronic condition lasting for at least six months, and hospitalization not related to COVID.

### Handling of missing data

To control for nonparticipation and attrition bias, and to take the EpiCov study design into account, we used the survey package^43^ on R software (version 4.1.0)^44^ and weights derived from nonparticipation models and calibration on margins, designed specifically for the EpiCov second follow-up, in all our analyses.

To control for item nonresponse among participants at baseline as well as at two follow-ups, we performed multiple imputation by chained equations (MICE) with 20 imputed data sets using the mice package^45^ on R software (version 4.1.0).^44^ Item nonresponse led to 14 614 complete cases and 3402 incomplete cases in the current analysis. Incomplete cases had missing data on the period of acute COVID (1.2% subjects eligible in the current analysis), on the presence of post-COVID symptoms (6.2% subjects infected during the first period; 3.1% subjects infected during the second period), or on covariates. Covariates with the highest missing rates were professional status (3.1%) and history of SARS-Cov-2 symptoms among household members (2.5%) for the first and second periods, respectively.

We included all variables of interest in the imputation model, as well as the survey weights^46^ and auxiliary variables that were associated with the variables of interest. We used the Classification and Regression Trees (CART) method, which automatically takes into account interactions between predictors thanks to recursive partitioning,^47^^,^^48^ to impute the missing observations for post-COVID symptoms, and the polytomous or binary logistic regression method for all other missing observations. For more details on the imputation model, see Appendix S1. We reproduced all our analyses on complete cases in a sensitivity analysis.

### Statistical analysis

First, we estimated the probabilities of six-month post-COVID symptoms for each period of acute COVID, as well as their 95% confidence intervals (CIs) using logit transformation. We then estimated the crude and adjusted relative risks (RRs and aRRs) of post-COVID symptoms according to the period of acute infection, with their 95% confidence intervals, using Poisson regressions with a robust variance estimator. We included all covariates cited previously in the multivariable analysis. We tested the null hypothesis of the regression coefficients being equal to zero with the Wald method.

Second, we assessed an effect modification by the period of acute COVID on sociodemographic or health-related risk factors for six-month post-COVID symptoms. As we hypothesized that the period of acute COVID could modify the effect of each risk factor on the risk of post-COVID symptoms, we conducted univariable and multivariable stratified Poisson regressions with a robust variance estimator separately for each period. We then tested the effect modification of the acute COVID period in a model gathering the two periods. To limit the number of tests in our study, we only tested interactions between the period and the variables which were independently associated with post-COVID symptoms in only one period, with visually different adjusted effect estimates between the two periods.

Because of variations in time lapses between the different EpiCov collection times (Figure 2), the duration of post-COVID symptoms considered in this study, initially set at six months, was, in practice, slightly shorter for subjects with a first acute COVID episode between mid-March and May than between mid-September and November 2020. As this difference could have led to underestimating the risk of six-month post-COVID symptoms in the second period, we reproduced the same analyses to compare the risk of post-COVID symptoms at strictly four weeks (instead of six months or more) after the onset of acute COVID between the two periods in a sensitivity analysis.

### Ethics

The French data protection authority, the ethics committee, and the “Comité du Label de la Statistique Publique” approved the survey in April 2020. All participants or their legally authorized representatives provided informed consent to participate in this study.

## Results

Among 82 616 subjects who were eligible for the current analysis, 10 201 and 6828 respectively had a first acute COVID during the first (mid-March-May 2020) and second (mid-September-November 2020) epidemic waves. The flow-case schedule is available in Figure S1.

The risk of 6-month post-COVID symptoms was higher when the acute COVID episode occurred during the first wave (14.6%; 95% CI, 13.9%-15.3%, Table 1) than when it occurred during the second wave (7.0%; 95% CI, 6.3%-7.7%), with an adjusted RR of 1.36 (95% CI, 1.20-1.55). The sensitivity analysis showed similar results when we considered post-COVID symptoms at four weeks after acute COVID (18.2% vs 8.6%; aRR 1.41; 95% CI, 1.26, 1.57; Table 1).

**Table 1:** Risk of post-COVID symptoms depending on the period of the first acute COVID episode EpiCov French national cohort, 2020-2021.

**Main analysis**	**Post-COVID symptoms ≥6 months**			
	**% of events**	**Crude RR**	**Adjusted RR**	
**Period of first acute COVID**	**(95% CI)** ^**a**^	**(95% CI)** ^**a**^	**(95% CI)** ^**a**^ ^**,**^ ^**b**^	***P* value** ^**a**^
First wave (mid-March to May 2020)	14.6 (13.9-15.3)	2.09 (1.83-2.39)	1.36 (1.20-1.55)	<0.001
Second wave (mid-September to November 2020)	7.0 (6.3-7.7)	1 (ref)	1 (ref)	
**Sensitivity analysis**	**Post-COVID symptoms at 4 weeks**			
**Period of first acute COVID**	**% of events**	**Crude RR**	**Adjusted RR**	***P* value** ^**a**^
	**(95% CI)** ^**a**^	**(95% CI)** ^**a**^	**(95% CI)** ^**a**^ ^**,**^ ^**b**^	
First wave (mid-March to May 2020)	18.2 (17.5-18.9)	2.11 (1.89-2.37)	1.41 (1.26-1.57)	<0.001
Second wave (mid-September to November 2020)	8.6 (7.9-9.3)	1 (ref)	1 (ref)	

Abbreviations: CI, confidence interval; RR, relative risk.

^a^Percentages, crude and adjusted relative risks, confidence intervals and two-sided *P* values were calculated on imputed data sets taking account of the EpiCov sampling design. Percentages were weighted by inverse inclusion probabilities, corrected for nonresponse and calibrated on the margin of census. Crude and adjusted relative risks were estimated using modified Poisson regressions. Two-sided *P* values were calculated on adjusted models and estimated via Wald tests.

^b^Relative risks are adjusted on gender, age, educational level, migratory status, professional status, deprivation status, age of the youngest child in the household, any history of SARS-Cov-2 symptoms among members of the household, presence of chronic mental condition(s), presence of chronic physical condition(s), body mass index, number of acute symptoms, COVID-related hospitalization, and non-COVID related hospitalization.

Some sociodemographic characteristics were found to be independently associated with post-COVID symptoms only when the first acute COVID episode occurred during the first epidemic wave (Table 2): being a woman (aRR 1.33; 95% CI, 1.16-1.54) and educational level (aRR for vocational diploma vs master’s degree: 1.37; 95% CI, 1.12-1.69). When the acute COVID episode occurred during the second wave, the effect estimates were lower for both variables, and their CIs included the null. No independent association was found for other sociodemographic characteristics, either during the first or the second wave. We found no effect modification by period of acute COVID for gender (*P = .*18) or for educational level (*P = .*18; Table 2).

**Table 2:** Risk of post-COVID symptoms in relation to sociodemographic characteristics, according to the period of first acute COVID EpiCov French national cohort, 2020-2021.

	**Acute COVID during the first epidemic wave (Mid-March-May 2020)**				**Acute COVID during the second epidemic wave (Mid-Sept-Nov 2020)**			
**Characteristics** ^**a**^	**% of events**	**Crude RR**	**Adjusted RR**		**% of events**	**Crude RR**	**Adjusted RR**	
	**(95 % CI)** ^**a**^	**(95% CI)** ^**a**^	**(95% CI)** ^**a**^ ^**,**^ ^**b**^	**P val** ^**a**^ ^**,**^ ^**b**^	**(95 % CI)** ^**a**^	**(95% CI)** ^**a**^	**(95% CI)** ^**a**^ ^**,**^ ^**b**^	***P* val** ^**a**^ ^**,**^ ^**b**^
Gender^c^				<0.001				0.35
Men	11.0 (10.5-11.4)	1 (ref)	1 (ref)		6.2 (5.9-6.4)	1 (ref)	1 (ref)	
Women	17.5 (17.2-17.8)	1.60 (1.37-1.86)	1.33 (1.16-1.54)		7.6 (7.3-8.0)	1.24 (0.98-1.56)	1.11 (0.89-1.39)	
Age^d^				0.58				0.45
15-24 years	9.4 (9.0-9.9)	1 (ref)	1 (ref)		5.7 (5.3-6.2)	1 (ref)	1 (ref)	
25-34 years	12.1 (11.7-12.4)	1.28 (0.97-1.67)	1.07 (0.80-1.44)		5.4 (5.0-5.7)	0.93 (0.60-1.44)	0.84 (0.51-1.38)	
35-44 years	15.1 (14.4-15.8)	1.60 (1.24-2.06)	1.12 (0.83-1.50)		6.8 (6.4-7.2)	1.18 (0.81-1.72)	1.16 (0.72-1.86)	
45-54 years	17.7 (17.2-18.1)	1.87 (1.46-2.39)	1.18 (0.90-1.55)		8.1 (7.5-8.8)	1.41 (0.97-2.06)	1.11 (0.72-1.72)	
55-64 years	16.7 (16.2-17.1)	1.77 (1.35-2.31)	1.18 (0.92-1.53)		9.2 (8.7-9.7)	1.61 (1.09-2.36)	1.25 (0.85-1.86)	
65 years old or older	17.3 (16.6-18.0)	1.83 (1.38-2.43)	1.31 (0.97-1.78)		8.1 (7.5-8.7)	1.41 (0.93-2.14)	1.32 (0.87-2.02)	
Educational level^c^				0.026				0.77
≥ Master’s degree	10.9 (10.5-11.3)	1 (ref)	1 (ref)		7.2 (6.8-7.6)	1 (ref)	1 (ref)	
> High school	13.5 (13.3-13.8)	1.24 (1.04-1.47)	1.15 (0.97-1.36)		7.2 (6.9-7.5)	1.00 (0.75-1.33)	0.91 (0.68-1.22)	
Vocational diploma	19.2 (18.6-19.8)	1.76 (1.43-2.17)	1.37 (1.12-1.69)		7.5 (7.1-7.8)	1.04 (0.72-1.49)	0.85 (0.59-1.21)	
≤ High school	16.3 (15.5-17.1)	1.49 (1.16-1.91)	1.25 (0.97-1.62)		6.0 (5.1-6.8)	0.83 (0.55-1.23)	0.81 (0.50-1.29)	
Migratory status^c^				0.40				0.12
Majority population	14.5 (14.3-14.8)	1 (ref)	1 (ref)		7.2 (7.0-7.5)	1 (ref)	1 (ref)	
*Non-European immigrants*								
First generation	12.6 (11.0-14.2)	0.87 (0.61-1.22)	0.75 (0.54-1.04)		2.9 (1.9-3.9)	0.39 (0.18-0.83)	0.40 (0.20-0.82)	
Second generation	14.5 (12.7-16.3)	1.00 (0.72-1.38)	0.96 (0.70-1.30)		7.5 (6.6-8.5)	1.03 (0.58-1.83)	1.08 (0.60-1.94)	
*European immigrants*								
First generation	14.1 (11.2-17.0)	0.97 (0.59-1.58)	0.92 (0.57-1.49)		7.5 (4.9-10.1)	1.02 (0.41-2.57)	0.83 (0.36-1.92)	
Second generation	18.1 (17.1-19.1)	1.25 (0.93-1.68)	1.15 (0.87-1.51)		7.5 (7.1-8.0)	1.04 (0.62-1.75)	1.04 (0.64-1.71)	
Professional status^c,e^				0.071				0.71
Non-workers	15.6 (15.1-16.2)	1 (ref)	1 (ref)		6.5 (6.0-6.9)	1 (ref)	1 (ref)	
Healthcare workers	20.4 (19.7-21.1)	1.31 (1.01-1.68)	1.29 (0.98-1.69)		9.4 (8.7-10.1)	1.45 (0.91-2.32)	1.25 (0.77-2.04)	
Other frontline workers	12.4 (11.2-13.7)	0.80 (0.60-1.06)	0.86 (0.64-1.15)		7.8 (6.8-8.8)	1.20 (0.75-1.93)	1.27 (0.79-2.03)	
Non-frontline workers	13.7 (13.2-14.2)	0.88 (0.75-1.03)	1.06 (0.87-1.29)		7.0 (6.7-7.4)	1.09 (0.85-1.40)	1.12 (0.81-1.55)	
Deprivation status^c^				0.47				0.23
Majority population	14.4 (14.2-14.7)	1 (ref)	1 (ref)		7.3 (7.1-7.5)	1 (ref)	1 (ref)	
Under poverty threshold	15.2 (14.5-15.9)	1.05 (0.85-1.31)	0.92 (0.75-1.14)		4.8 (4.1-5.4)	0.65 (0.45-0.96)	0.80 (0.56-1.16)	
Youngest child age^c^				0.38				0.85
No child	13.2 (12.9-13.4)	1 (ref)	1 (ref)		7.1 (6.9-7.4)	1 (ref)	1 (ref)	
<6 years	13.6 (12.7-14.4)	1.03 (0.83-1.28)	1.05 (0.83-1.33)		5.6 (5.2-5.9)	0.78 (0.57-1.08)	0.91 (0.61-1.37)	
6-12 years	18.3 (17.6-18.9)	1.38 (1.12-1.71)	1.25 (1.00-1.57)		6.0 (5.3-6.8)	0.85 (0.58-1.23)	0.79 (0.53-1.20)	
>12 years	17.7 (17.1-18.3)	1.34 (1.08-1.67)	1.11 (0.90-1.38)		9.1 (8.2-10.1)	1.28 (0.90-1.83)	0.98 (0.68-1.43)	
Lives alone	16.0 (15.4-16.5)	1.21 (0.99-1.49)	1.07 (0.87-1.31)		7.2 (6.5-7.9)	1.01 (0.72-1.43)	0.96 (0.69-1.34)	
Symptomatic relative^d^				0.99				0.95
No	13.5 (13.2-13.8)	1 (ref)	1 (ref)		6.6 (6.3-6.9)	1 (ref)	1 (ref)	
Yes	16.6 (15.9-17.3)	1.23 (1.06-1.42)	1.00 (0.87-1.16)		7.8 (7.5-8.2)	1.19 (0.95-1.50)	0.99 (0.78-1.27)	

Abbreviations: CI, confidence interval; RR, relative risk.

^a^Percentages, crude and adjusted relative risks, confidence intervals and two-sided *P* values were calculated taking the EpiCov were calculated on imputed data sets taking account of the EpiCov sampling design. Percentages were weighted by inverse inclusion probabilities, corrected for nonresponse, and calibrated on the margin of census. Crude and adjusted relative risks were estimated using modified Poisson regressions. Two-sided *P* values were calculated on adjusted models and estimated via Wald tests. *P* values were calculated on adjusted models and were estimated via Wald tests.

^b^Multivariable analysis: variables are mutually adjusted, and adjusted for and adjusted for physical or mental chronic mental conditions at baseline, body mass index, number of acute symptoms, COVID-related and non COVID-related hospitalization.

^c^Information collected at baseline.

^d^Information collected when acute COVID symptoms were reported.

Considering health-related characteristics (Table 3) during the first wave, the risk of six-month post-COVID symptoms was found to be higher of the presence of physical (aRR 1.40; 95% CI, 1.21-1.61) or mental (aRR 1.37; 95% CI, 1.14, 1.64) condition(s) at baseline and to increase with the number of acute symptoms in univariable, as well as in multivariable analysis. During the second epidemic wave, similar associations were observed between the risk of post-COVID symptoms and the presence of any physical condition at baseline (aRR 1.57; 95% CI, 1.25-1.96), and between the risk of post-COVID symptoms and a high number of acute symptoms. However, no association was found between the risk of post-COVID symptoms and the presence of any mental condition at baseline (aRR 1.12; 95% CI, 0.80-1.57). No independent association was found between post-COVID symptoms and BMI, or between post-COVID symptoms and any history of hospitalization during the pandemic. We found no effect modification by period of acute COVID for the presence of any mental condition at baseline (*P = .*26; Table 3).

**Table 3:** Risk of post-COVID symptoms in relation to socio-health-related characteristics, according to the period of first acute COVID EpiCov French national cohort, 2020-2021.

	**Acute COVID during the first epidemic wave (Mid-March-May 2020)**				**Acute COVID during the second epidemic wave (Mid-Sept- Nov 2020)**			
**Characteristics** ^**a**^	**% of events**	**Crude RR**	**Adjusted RR**		**% of events**	**Crude RR**	**Adjusted RR**	
	**(95 % CI)** ^**a**^	**(95% CI)** ^**a**^	**(95% CI)** ^**a**^ ^**,**^ ^**b**^	**P val** ^**a**^ ^**,**^ ^**b**^	**(95 % CI)** ^**a**^	**(95% CI)** ^**a**^	**(95% CI)** ^**a**^ ^**,**^ ^**b**^	***P* val** ^**a**^ ^**,**^ ^**b**^
Baseline physical illness(es)^c^				<0.001				<0.001
No	11.1 (10.8-11.3)	1 (ref)	1 (ref)		5.7 (5.4-5.9)	1 (ref)	1 (ref)	
Yes	19.8 (19.4-20.3)	1.80 (1.57-2.05)	1.40 (1.21-1.61)		9.7 (9.2-10.1)	1.71 (1.36-2.14)	1.57 (1.25-1.96)	
Baseline mental illness(es)^c^				<0.001				0.52
No	13.0 (12.8-13.3)	1 (ref)	1 (ref)		6.7 (6.5-7.0)	1 (ref)	1 (ref)	
Yes	27.6 (26.6-28.7)	2.12 (1.80-2.50)	1.37 (1.14-1.64)		10.0 (9.0-11.0)	1.49 (1.07-2.07)	1.12 (0.80-1.57)	
BMI^d^				0.057				0.93
≤25	12.5 (12.1-12.8)	1 (ref)	1 (ref)		6.8 (6.5-7.1)	1 (ref)	1 (ref)	
>25	17.1 (16.9-17.4)	1.38 (1.20-1.58)	1.14 (1.00-1.29)		7.2 (6.9-7.6)	1.07 (0.85-1.33)	1.01 (0.80-1.28)	
Number of acute symptoms^d^				<0.001				<0.001
3 or fewer	5.6 (5.4-5.9)	1 (ref)	1 (ref)		3.8 (3.7-4.0)	1 (ref)	1 (ref)	
4-5	13.9 (13.4-14.4)	2.48 (2.01-3.05)	2.40 (1.96-2.94)		9.9 (9.2-10.6)	2.58 (1.87-3.57)	2.70 (1.96-3.72)	
6-7	24.0 (23.4-24.6)	4.27 (3.50-5.23)	3.99 (3.28-4.85)		16.9 (15.7-18.1)	4.39 (3.30-5.85)	4.45 (3.35-5.91)	
8 or more	41.4 (40.5-42.3)	7.38 (6.15-8.87)	6.40 (5.30-7.73)		29.7 (28.2-31.2)	7.75 (5.92-10.14)	7.33 (5.55-9.67)	
Hospitalized for COVID^d^				0.27				0.89
No	14.2 (14.0-14.4)	1 (ref)	1 (ref)		6.8 (6.6-7.1)	1 (ref)	1 (ref)	
Yes	32.3 (25.2-39.5)	2.27 (1.51-3.41)	1.25 (0.85-1.85)		13.2 (9.1-17.2)	1.91 (0.92-3.93)	1.05 (0.52-2.14)	
Other hospitalization				0.63				0.56
No	14.5 (14.3-14.8)	1 (ref)	1 (ref)		6.8 (6.6-7.1)	1 (ref)	1 (ref)	
Yes	17.1 (13.8-20.4)	1.17 (0.66-2.08)	0.86 (0.46-1.61)		10.2 (8.1-12.3)	1.49 (0.87-2.55)	1.17 (0.69-1.97)	

Abbreviations: BMI, body mass index; CI, confidence interval; RR, relative risk.

^a^Percentages, crude and adjusted relative risks, confidence intervals and two-sided *P* values were calculated on imputed data sets taking the EpiCov sampling design into account. Percentages were weighted by inverse inclusion probabilities, corrected for nonresponse, and calibrated on the margin of census. Crude and adjusted relative risks were estimated using modified Poisson regressions. Two-sided *P* values were calculated on adjusted models and estimated via Wald tests. *P* values were calculated on adjusted models and were estimated via Wald tests.

^b^Multivariable analysis: variables are mutually adjusted, and adjusted for gender, age, educational level, migratory status, professional status, deprivation status, age of the youngest child in the household, and any history of SARS-Cov-2 symptoms among members of the household.

^c^Information collected at baseline.

^d^Information collected when acute COVID symptoms were reported.

We observed higher risks of six-month post-COVID symptoms when the acute COVID occurred during the first wave within each level of the sociodemographic (Table 2) and health-related (Table 3) variables of interest, as well as across the whole study population (Table 1). In the complete case analysis, the risk of six-month post-COVID symptoms remained higher during the first rather than during the second epidemic wave (14.3% vs 6.7%, see Table S1), with a seemingly higher adjusted effect estimate than in the main analysis (aRR 1.75; 95% CI, 1.53-2.00). We found no major difference with the main analysis regarding independent sociodemographic and health-related risk factors for post-COVID symptoms, either for the first or the second wave (see Tables S2 and S3).

## Discussion

In this random prospective cohort representative of the general population aged 15 or older in France, the risk of six-month post-COVID symptoms was 14.6% (95% CI, 13.9%-15.3%, Table 1) when the first acute COVID episode occurred during the first epidemic wave (between mid-March and May 2020) vs 7.0% (95% CI, 6.3%-7.7%) when it occurred during the second epidemic wave (between mid-September and November 2020). The risk of post-COVID symptoms remained higher for the first wave after adjustment for health-related and sociodemographic characteristics (aRR 1.36; 95% CI, 1.20-1.55). As noted in the whole study population, we observed a lower risk of post-COVID symptoms during the second rather than during the first epidemic wave within each category of sociodemographic or health-related covariates considered in this study.

For both periods, in multivariable analysis, the risk of post-COVID symptoms increased with the number of acute symptoms, and it was higher in the presence of physical condition(s) at baseline. Moreover, during the first wave, the risk of post-COVID symptoms was found to be higher in women, in the presence of baseline mental condition(s), and to vary with educational level.

### Findings in context

#### Risk of post-COVID symptoms according to the period of acute COVID

In this study, the lower risk of six-month post-COVID symptoms observed for the second compared to the first wave cannot be linked to the spread of SARS-Cov-2 variants^37^ nor to the availability of anti-SARS-Cov-2 vaccines,^38^ which occurred at the very end of 2020 in France. In addition, it is not likely to be linked to changes in sociodemographic or health profiles of infected subjects between the two periods as we observed the risk reduction, in the whole study population, after adjustment for a high number of recognized risk factors for post-COVID symptoms, and, in stratified analysis, within each level of these risk factors. Such results suggest that the medical uncertainty, or the consequences of stringent preventive measures, that characterized the early pandemic, played a role in the risk of post-COVID symptoms.

These results are in line with a French study conducted using a convenience sample of subjects with post-COVID symptoms in August 2021. The proportion of subjects with a history of SARS-Cov-2 infection during the French first epidemic wave (before June 2020) was higher among the participants than in subjects with a history of SARS-Cov-2 infection in the general population (odds ratio 1.82; 95% CI, 1.55-2.15).^33^ Our study, using data from a large prospective cohort representative of the general population, corroborates these results at a national level. It also suggests that the higher risk of post-COVID symptoms during the first wave cannot be explained by a subsequent change in health-related or sociodemographic profiles of infected subjects.

#### Risk factors for post-COVID symptoms

In this study, independent sociodemographic and health-related risk factors for post-COVID symptoms were consistent with the literature^7^^,^^15^^,^^17^: having a high number of acute symptoms and chronic physical condition(s) at baseline for both periods of acute COVID; being a woman and having mental condition(s) at baseline for the first wave. The association found between educational level and post-COVID symptoms during the first wave is also in line with studies showing a higher risk of post-COVID symptoms in case of low socioeconomic status.^17^^,^^49^

### Strengths and limitations

To our knowledge, this study is the first to give insight into temporal changes in the risk of post-COVID symptoms and in their associated factors, in general population and at a national level, before the arrival of variants and before the availability of anti-SARS-Cov-2 vaccines. We estimated this risk from the French national socioepidemiologic EpiCov cohort, using a probability sampling design, providing a large random sample selected from a national administrative database covering more than 96% of the French population. In addition, our analysis focused on symptoms present at six months or more after acute COVID, whereas most studies have so far focused on symptoms lasting less than six months.^14^ Lastly, the ECDC definition of acute COVID, although based on self-reported symptoms, showed good specificity when we used SARS-Cov-2 serology obtained from home capillary blood self-sampling (ELISA-S, Euroimmun) for detection of anti-SARS-Cov-2 antibodies (IgG) against the S1 domain of the viral spike protein as reference standard: 88.2% (95% CI, 87.1%-89.1%) in the subsample of 9032 subjects with a SARS-Cov-2 serology at baseline (May 2020), and 90.4% in the 55 357 (95% CI, 90.0%-90.8%) subjects with a SARS-Cov-2 serology at first follow-up.

This study has several limitations. First, post-COVID symptoms considered here were mainly physical symptoms. In particular, we did not consider cognitive dysfunction, which is one of the most frequent post-COVID symptoms.^3^ Therefore, we may have underestimated the risk of post-COVID symptoms in this study. Nonetheless, we considered persistent fatigue, which is strongly correlated with post-COVID cognitive dysfunction.^3^^,^^50^

Second, we restricted our analysis to participants who had a first acute COVID between mid-March and May reported at baseline (May 2020), or a first acute COVID between mid-September and November reported at first follow-up (November 2020). The choice of the first period was linked to the time frame of EpiCov questionnaire: at baseline, participants were asked to report symptoms present after mid-March (thus excluding symptoms resolved before this date). The choice of the second period was made to ensure that the maximum time lapse between the onset and the reporting of acute symptoms was comparable between the two exposure groups. Therefore, although most SARS-Cov-2 infections occurred in France during the two periods studied here (Figure 2), we cannot exclude unobserved temporal changes in the risk of post-COVID symptoms before mid-March 2020, or between May and mid-September 2020.

Third, nonparticipation in EpiCov baseline or follow-ups, as well as item nonresponse among participants, might have limited the external validity of the results. However, selection and attrition bias, usual in studies conducted in general population, were limited by the implementation of nonparticipation adjustment weights, obtained from nonresponse models and calibration on margins derived from the census. We used the weights that were designed specifically for EpiCov second follow-up, using demographic and socioeconomic indicators available for all eligible subjects in the sampling frame, and described elsewhere.^35^ Moreover, we used MICE to handle item nonresponse. MICE relies on the missing at random (MAR) assumption, which can hardly be evaluated using observed data.^51^ However, we included variables in the imputation model that were associated with variables of interest or potentially related to missing data mechanisms, which increases the plausibility of MAR assumptions.^52^ We found a higher risk of post-COVID symptoms during the second wave, both after multiple imputation and in the complete case analysis. However, the adjusted effect estimate for the period seemed lower after multiple imputation (aRR 1.36; 95% CI, 1.20-1.55) than in the complete case analysis (aRR 1.75; 95% CI, 1.53-2.00). This difference could be due to the inclusion of health-related auxiliary variables, that enabled us to account for differential nonresponse mechanisms between the 2 groups, in the imputation model. Indeed, subjects with an acute COVID during the second wave had more frequently missing data on post-COVID symptoms if they reported any new chronic condition at second follow-up (see Table S4). This association was not found among subjects infected during the first wave.

Last, in our study, the duration of post-COVID symptoms, initially set at six months, was in practice shorter for subjects with an acute COVID during the first rather than the second wave. Therefore, we potentially underestimated the risk of post-COVID symptoms in the second period. However, when we considered the risk of post-COVID symptoms at strictly 4 weeks, the effect estimates of acute period were similar to the ones we found in the main analysis (18.2% vs 8.6%, aRR 1.41; 95% CI, 1.26-1.57).

### Perspectives

There is evidence that post-COVID symptoms could last for years. Further analyses will be conducted among EpiCov participants who were infected during the first or the second wave to estimate their risk of post-COVID symptoms at 1 year or more, using data from the second (July 2021) and third (October 2022) follow-ups. These follow-ups will also offer the opportunity to estimate the risk of post-COVID symptoms according to the type of SARS-Cov-2 variant contracted and/or the vaccination history of subjects infected after 2020.

In this national random population-based cohort, we observed a higher risk of six-month post-COVID symptoms when the acute COVID occurred during the first wave (March-May 2020) rather than during the second epidemic wave (September-November 2020) in mainland France. This study, conducted before the spread of SARS-Cov-2 variants and the availability of vaccines, suggests that the medical uncertainty and/or the consequences of stringent preventive measures, which characterized the beginning of the pandemic, played a role in the risk of post-COVID symptoms.

## Acknowledgments

The authors would like to thank the members of the EpiCov study group, who all devoted a significant amount of work to make this study possible. We warmly thank the National Institute of Health and Medical Research staff who worked with considerable dedication and commitment to make it possible to develop, in record time, and to maintain all regulatory, budgetary, technical, and logistical aspects of the EpiCov study. We warmly thank the staff of the French Public Health Agency who played a major role in organization and quality assurance for the seroprevalence component of the EpiCov study. We thank the staff of the Directorate for Research, Studies, Evaluation and Statistics and the French National Institute for Statistics and Economic Studies, for their collaboration in the implementation of the study, methodological input, sample selection, and the complex development of weights to correct for nonresponse . We thank the staff of the *Institut Public de Sondage d’Opinion Secteur* for their major contribution to the quality of data collection. We thank the Biological Resource Center biobanks staff, and especially their heads, Dr. Isabelle Pellegrin, and Julien Jeanpetit (Robert Pellegrin French University Hospital, Bordeaux, France), Edouard Tuaillon (Biological Resources Center of the French University Hospital of Montpellier), Dr. Yves-Edouard Herpe (Biological Resource Center biobank of Picardie), Jacqueline Deloumeaux (Biological Resource Center of the French University Hospital of Guadeloupe), Dr. Rémi Neviere (Biological Resource Center of Martinique), Julien Eperonnier, Estelle Nobecourt (Biological Resource Center of the Réunion) for the quality of sample management of the EpiCov study. We thank the biobank team in the National Institute of Health and Medical Research SC10. We also thank the staff of the Emerging Viruses Unit for the high-quality management of such a large number of serological assays. We also warmly thank Stef van Buuren, who developed the MICE package on R software with Dr. Karin Groothuis-Oudshoorn, and who kindly answered our questions regarding methods of multiple imputation.

## Joint authorship

Members of the EpiCov study group: Dr. Josiane Warszawski (principal investigator and scientific codirector), Dr. Nathalie Bajos (scientific codirector), Guillaume Bagein, Dr. François Beck, Dr. Emilie Counil, Dr. Florence Jusot, Dr. Nathalie Lydié, Dr. Claude Martin, Dr. Laurence Meyer, Dr. Philippe Raynaud, Dr. Alexandra Rouquette, Dr. Ariane Pailhé, Dr. Delphine Rahib, Dr. Patrick Sillard, Dr. Rémy Slama, and Dr. Alexis Spire.

## Supplementary Material

Web_Material_kwae174

## Data Availability

The EpiCov study data are available for research purposes after submission for approval by the French Ethics and Regulatory Committee (Confidentiality Committee, Ethical and Scientific Committee for Research, Studies and Evaluations in the Field of Health and National Data Protection Commission). The access procedure is available on the Secure Data Access Centre (https://www.casd.eu/). Additional information, as well as the codes used for the study analysis, can be requested from the corresponding author.
