# Supplementary material for: Temporal changes in the risk of six-month post-COVID symptoms: a national population-based cohort study
Source: Am J Epidemiol. 2024 Jul 3;194(1):162–71. doi: 10.1093/aje/kwae174 (PMC11735949; doi:10.1093/aje/kwae174)
Supplement: Web_Material_kwae174 [file web_material_kwae174.docx]

***Supplementary Data***

**Temporal changes in the risk of six-month post-covid symptoms:**

**a national population-based cohort study**

**Authors:** Anne Pastorello, Laurence Meyer, Joël Coste, Camille Davisse-Paturet, Xavier de Lamballerie, Maria Melchior, Sophie Novelli, Delphine Rahib, Nathalie Bajos, Cécile Vuillermoz, Jeanna-Ee Franck, Carmelite Manto, Alexandra Rouquette, Josiane Warszawski, for the EpiCov study group

**Table of contents**

**Appendix S1:** Variables used for Multiple Imputation by Chained Equations (page 2)

**Figure S1:** Case – flow schedule (page 3)

**Table S1:** Risk of post-covid symptoms depending on the period of the first acute covid episode -complete cases analysis (page 4)

**Table S2:** Risk of post-covid symptoms in relation to socio-demographic characteristics, according to the period of first acute covid – complete cases analysis (page 5)

**Table S3:** Risk of post-covid symptoms in relation to health-related characteristics, according to the period of first acute covid – complete cases analysis (page 8)

**Table S4:** Comparison of subjects with observed and missing data on post-covid symptoms regarding auxiliary health-related variables, stratified on the period (page 10)

**Appendix S1: Variables used for Multiple Imputation by Chained Equations**

We included all variables of interest (post-covid symptoms, period of acute covid, covariates), as well as the survey weights designed for EpiCov second-follow-up (Kim, Michael Brick, Fuller, Kalton 2006), in the imputation model. To enhance the plausibility of the missing at random (MAR) assumption, we also included a set of auxiliary variables in the imputation model. Auxiliary variables were shown to reduce the risk of biased estimates after multiple imputation, and to enhance the precision of estimations, provided that these variables are strongly associated with the variables of interest or related the missing data mechanisms, and that these auxiliary variables have few or no missing observations (Collins, Schafer, Kam 2001). We included auxiliary variables that were:

- Associated with socio-demographic covariates at baseline: household size, perceived financial situation;
- Associated with health state at baseline: smoking status, alcohol consumption;
- Associated with the presence of six-month post-covid symptoms at first or second follow-up: perceived health-state and any new chronic condition or disability within the last six months.

Auxiliary variables included in the imputation model had no more than 0.2 % missing data.

**Figure S1**


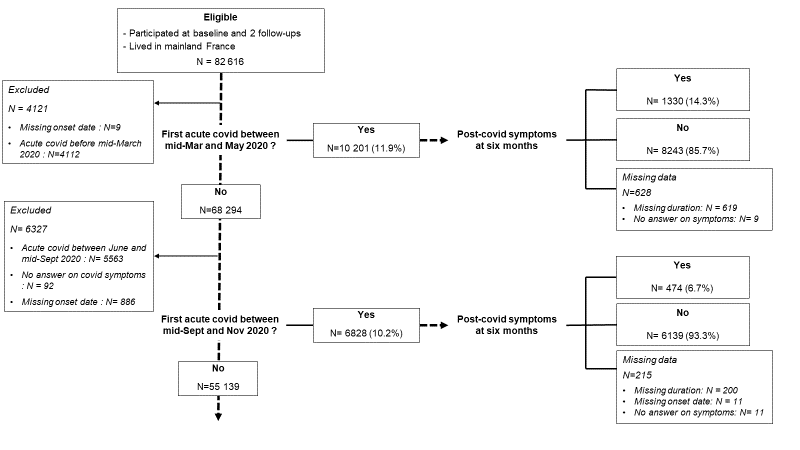
Case–flow schedule. EpiCov French national cohort (2020-2021). Percentages are weighted; numbers are unweighted. One subject may have missing data on acute covid or post-covid symptoms for several reasons.

| Table S1: Risk of post-covid symptoms depending on the period of the first acute covid episode – complete cases analysis | | | | | | |
| --- | --- | --- | --- | --- | --- | --- |
| *EpiCov French national cohort, 2020-2021* | | | | | |  |
| **Period of first acute covid** | | **No. events / no. complete cases^a^** | **% of events** | **Crude RR** | **Adjusted RR** | ***P* value^a^** |
|  |  |  | **(95% CI)^a^** | **(95% CI)^a^** | **(95% CI)^a,b^** |  |
|  | |  |  |  | ***N= 14 614*** |  |
|  | **First wave** (mid-March to May 2020), N= 10 201 | 1330 / 9573 | 14.3 (13.4, 15.3) | 2.13 (1.87, 2.43) | 1.75 (1.53, 2.00) | <0.001 |
|  | **Second wave** (mid-September to November 2020), N= 6828 | 474 / 6613 | 6.7 (6.0, 7.5) | 1 (ref) | 1 (ref) |  |
| Abbreviations: CI, Confidence Interval; RR, Relative Risk. | | | | | | |
| ^a^Percentages, crude and adjusted relative risks, confidence intervals and two-sided *P* values were calculated taking account of the EpiCov sampling design. Percentages were weighted by inverse inclusion probabilities, corrected for non-response and calibrated on the margin of census. Crude and adjusted relative risks were estimated using modified Poisson regressions. Two-sided *P* values were calculated on adjusted models and estimated via Wald tests. | | | | | | |
| ^b^ Relative risks are adjusted on gender, age, educational level, migratory status, professional status, deprivation status, age of the youngest child in the household, any history of SARS-Cov-2symptoms among members of the household, the presence of chronic mental condition(s), the presence of chronic physical condition(s), Body Mass Index, the number of acute symptoms, covid-related hospitalization, and non-covid related hospitalization. | | | | | | |

| **Table S2:** **Risk of post-covid symptoms in relation to socio-demographic characteristics, according to the period of first acute covid – complete cases analysis** | | | | | | | | | | | | | |
| --- | --- | --- | --- | --- | --- | --- | --- | --- | --- | --- | --- | --- | --- |
| *EpiCov French national cohort, 2020-2021* | | | | | | | | | | |  |  |  |
|  | | | | **Acute covid during the first wave (mid-March to May 2020), N= 10 201** | | | | | **Acute covid during the second wave (mid-Sept to Nov 2020), N= 6828** | | | | |
| **Characteristics^a^** | | | | **Total** | **Post-covid** | **Crude RR** | **Adjusted RR** | ***P* val^a,b^** | **Total** | **Post-covid** | **Crude RR** | **Adjusted RR** | ***P* val^a,b^** |
|  |  |  |  | **N^a^** | **symptoms %(n)^a^** | **(95% CI)^a^** | **(95% CI)^a,b^** |  | **N^a^** | **symptoms %(n)^a^** | **(95% CI)^a^** | **(95% CI)^a,b^** |  |
|  | | | |  |  |  | ***N= 8639*** |  |  |  |  | ***N= 5975*** |  |
| **Gender^c^** | | | |  |  |  |  | <0.001 |  |  |  |  | 0.68 |
| Men | | 3942 | 10.6 (403) | 1 (ref) | 1 (ref) |  | 2663 | 6.1 (171) | 1 (ref) | 1 (ref) |  |  |  |
| Women | | 5631 | 17.4 (927) | 1.63 (1.41, 1.90) | 1.40 (1.20, 1.63) |  | 3950 | 7.3 (303) | 1.20 (0.95, 1.50) | 1.05 (0.84, 1.32) |  |  |  |
| **Age^d^** | | | |  |  |  |  | 0.22 |  |  |  |  | 0.62 |
| 15-24 years | | 1226 | 9.3 (123) | 1 (ref) | 1 (ref) |  | 1256 | 5.3 (64) | 1 (ref) | 1 (ref) |  |  |  |
| 25-34 years | | 1499 | 12.0 (180) | 1.29 (0.98, 1.70) | 1.00 (0.73, 1.35) |  | 955 | 5.3 (54) | 1.00 (0.64, 1.55) | 0.82 (0.49, 1.39) |  |  |  |
| 35-44 years | | 2090 | 14.9 (287) | 1.60 (1.23, 2.08) | 0.99 (0.73, 1.36) |  | 1296 | 6.6 (97) | 1.24 (0.85, 1.81) | 0.99 (0.61, 1.59) |  |  |  |
| 45-54 years | | 2199 | 17.5 (358) | 1.88 (1.46, 2.43) | 1.17 (0.86, 1.58) |  | 1305 | 7.6 (107) | 1.42 (0.97, 2.08) | 1.03 (0.66, 1.60) |  |  |  |
| 55-64 years | | 1569 | 16.1 (222) | 1.73 (1.31, 2.29) | 1.23 (0.93, 1.63) |  | 1011 | 9.1 (85) | 1.70 (1.16, 2.50) | 1.18 (0.79, 1.78) |  |  |  |
| 65 years old or older | | 990 | 17.2 (160) | 1.85 (1.39, 2.46) | 1.35 (0.97, 1.88) |  | 790 | 7.9 (67) | 1.48 (0.99, 2.22) | 1.33 (0.85, 2.07) |  |  |  |
| **Educational level^c^** | | | |  |  |  |  | 0.081 |  |  |  |  | 0.44 |
| ≥ Master’s degree | | 2042 | 10.8 (233) | 1 (ref) | 1 (ref) |  | 1265 | 6.9 (87) | 1 (ref) | 1 (ref) |  |  |  |
| > High school | | 5217 | 13.3 (727) | 1.23 (1.04, 1.46) | 1.12 (0.94, 1.33) |  | 3437 | 7.0 (247) | 1.01 (0.76, 1.35) | 0.88 (0.66, 1.18) |  |  |  |
| Vocational diploma | | 1351 | 19.0 (235) | 1.76 (1.42, 2.18) | 1.30 (1.04, 1.63) |  | 954 | 7.4 (78) | 1.07 (0.75, 1.55) | 0.78 (0.54, 1.13) |  |  |  |
| ≤ High school | | 954 | 16.0 (131) | 1.48 (1.15, 1.91) | 1.30 (0.99, 1.71) |  | 953 | 5.5 (62) | 0.80 (0.55, 1.17) | 0.71 (0.44, 1.14) |  |  |  |
| **Migratory status^c^** | | | |  |  |  |  | 0.61 |  |  |  |  | 0.12 |
| Majority population | | 7927 | 14.1 (1088) | 1 (ref) | 1 (ref) |  | 5563 | 6.9 (407) | 1 (ref) | 1 (ref) |  |  |  |
| *Non-European immigrants* | | |  |  |  |  |  |  |  |  |  |  |  |
| First generation | | 392 | 12.7 (54) | 0.90 (0.64, 1.25) | 0.86 (0.62, 1.19) |  | 232 | 2.3 (9) | 0.33 (0.15, 0.73) | 0.33 (0.15, 0.76) |  |  |  |
| Second generation | | 444 | 14.1 (62) | 1.00 (0.72, 1.37) | 1.13 (0.83, 1.53) |  | 293 | 7.4 (18) | 1.06 (0.58, 1.93) | 1.00 (0.52, 1.92) |  |  |  |
| *European immigrants* | | |  |  |  |  |  |  |  |  |  |  |  |
| First generation | | 197 | 12.5 (24) | 0.89 (0.55, 1.43) | 0.77 (0.46, 1.30) |  | 95 | 6.5 (6) | 0.93 (0.35, 2.51) | 0.99 (0.45, 2.14) |  |  |  |
| Second generation | | 487 | 17.7 (76) | 1.25 (0.92, 1.72) | 1.07 (0.81, 1.41) |  | 322 | 7.9 (24) | 1.13 (0.67, 1.91) | 1.18 (0.70, 1.99) |  |  |  |
| **Professional status^c, e^** | | | |  |  |  |  | 0.026 |  |  |  |  | 0.81 |
| Non-workers | | 2497 | 15.1 (347) | 1 (ref) | 1 (ref) |  | 2108 | 6.3 (140) | 1 (ref) | 1 (ref) |  |  |  |
| Healthcare workers | | 562 | 20.5 (109) | 1.36 (1.05, 1.77) | 1.36 (1.02, 1.83) |  | 364 | 9.0 (30) | 1.43 (0.88, 2.34) | 1.20 (0.72, 2.02) |  |  |  |
| Other frontline workers | | 756 | 11.5 (98) | 0.76 (0.58, 1.00) | 0.85 (0.63, 1.15) |  | 428 | 7.2 (32) | 1.15 (0.71, 1.88) | 1.04 (0.62, 1.75) |  |  |  |
| Non-frontline workers | | 5470 | 13.4 (727) | 0.89 (0.75, 1.05) | 1.08 (0.87, 1.35) |  | 3548 | 6.8 (260) | 1.09 (0.85, 1.40) | 1.15 (0.85, 1.57) |  |  |  |
| **Deprivation status^c^** | | | |  |  |  |  | 0.11 |  |  |  |  | 0.069 |
| Majority population | | 8535 | 14.3 (1183) | 1 (ref) | 1 (ref) |  | 5936 | 7.0 (426) | 1 (ref) | 1 (ref) |  |  |  |
| Under poverty threshold | | 1038 | 14.6 (147) | 1.03 (0.82, 1.29) | 0.82 (0.64, 1.04) |  | 677 | 4.6 (48) | 0.65 (0.45, 0.94) | 0.69 (0.46, 1.03) |  |  |  |
| **Youngest child age** | | |  |  |  |  |  |  |  |  |  | 0.79 |  |
| No child | | 4784 | 12.9 (634) | 1 (ref) | 1 (ref) | 0.17 | 3608 | 6.9 (244) | 1 (ref) | 1 (ref) |  |  |  |
| <6 years | | 1436 | 13.4 (173) | 1.04 (0.85, 1.28) | 1.16 (0.91, 1.48) |  | 906 | 5.4 (63) | 0.79 (0.57, 1.10) | 0.97 (0.64, 1.47) |  |  |  |
| 6-12 years | | 1040 | 18.3 (165) | 1.42 (1.15, 1.76) | 1.34 (1.06, 1.69) |  | 714 | 5.7 (48) | 0.86 (0.58, 1.19) | 0.81 (0.53, 1.24) |  |  |  |
| >12 years | | 990 | 17.6 (164) | 1.36 (1.09, 1.71) | 1.06 (0.84, 1.36) |  | 647 | 8.6 (58) | 1.25 (0.87, 1.80) | 1.04 (0.70, 1.55) |  |  |  |
| Lives alone | | 1321 | 15.8 (194) | 1.22 (0.99, 1.51) | 1.12 (0.90, 1.40) |  | 737 | 7.0 (61) | 1.02 (0.72, 1.43) | 0.89 (0.64, 1.24) |  |  |  |
| **Potentially infected relative** | | |  |  |  |  |  |  |  |  |  | 0.65 |  |
| No | | 6152 | 13.3 (799) | 1 (ref) | 1 (ref) | 0.87 | 4376 | 6.3 (297) | 1 (ref) | 1 (ref) |  |  |  |
| Yes | | 3165 | 16.5 (488) | 1.24 (1.07, 1.43) | 0.99 (0.85,1.15) |  | 2064 | 7.6 (163) | 1.20 (0.94, 1.52) | 0.94 (0.73, 1.22) |  |  |  |
| Abbreviations: CI, Confidence Interval; RR, Relative Risk. | | | | | | | | | | | | | |
| ^a^Percentages, crude and adjusted relative risks, confidence intervals and two-sided *P* values were calculated taking the EpiCov sampling design into account. Percentages were weighted by inverse inclusion probabilities, corrected for non-response and calibrated on the margin of census. Crude and adjusted relative risks were estimated using modified Poisson regressions. Two-sided *P* values were calculated on adjusted models and estimated via Wald tests. *P* values were calculated on adjusted models and were estimated via Wald tests. | | | | | | | | | | | | | |
| **^b^**Multivariable analysis: variables are mutually adjusted, and adjusted for physical or mental chronic mental conditions at baseline, Body Mass Index, number of acute symptoms, covid-related and non covid-related hospitalization. | | | | | | | | | | | | | |
| **^c^**Information collected at baseline. | | | | | | | | | | | | | |
| **^d^**Information collected when acute covid symptoms were reported. | | | | | | | | | | | | | |
| **^e^**Healthcare workers: medical and paramedical staff, firemen, first aid, paramedics, pharmacists. Other frontline workers: Home helps or housekeepers, food shop workers, delivery, public transportation and cab drivers, bank customer services or reception staff, petrol station employees, police officers, postal workers, cleaning staff, security guards, construction workers, truck drivers, farmers and social workers. | | | | | | | | | | | | | |

| **Table S3: Risk of post-covid symptoms in relation to health-related characteristics, according to the period of first acute covid – complete cases analysis** | | | | | | | | | | | | | |
| --- | --- | --- | --- | --- | --- | --- | --- | --- | --- | --- | --- | --- | --- |
| *EpiCov French national cohort, 2020-2021* | | | | | | | | | | |  |  |  |
|  | | | | **Acute covid during the first wave (mid-March to May 2020), N= 10 201** | | | | | **Acute covid during the second wave (mid-Sept to Nov 2020), N= 6828** | | | | |
| **Characteristics^a^** | | | | **Total** | **Post-covid** | **Crude RR** | **Adjusted RR** | ***P* val^a,b^** | **Total** | **Post-covid** | **Crude RR** | **Adjusted RR** | ***P* val^a,b^** |
|  |  |  |  | **N^a^** | **symptoms %(n)^a^** | **(95% CI)^a^** | **(95% CI)^a,b^** |  | **N^a^** | **symptoms %(n)^a^** | **(95% CI)^a^** | **(95% CI)^a,b^** |  |
|  | | | |  |  |  | ***N= 8639*** |  |  |  |  | ***N= 5975*** |  |
| **Baseline physical illness(es)^c^** | | | |  |  |  |  | 0.001 |  |  |  |  | 0.001 |
| No | | 5837 | 10.8 (656) | 1 (ref) | 1 (ref) |  | 4411 | 5.4 (259) | 1 (ref) | 1 (ref) |  |  |  |
| Yes | | 3536 | 19.4 (624) | 1.80 (1.56, 2.07) | 1.30 (1.12, 1.51) |  | 2051 | 9.3 (199) | 1.72 (1.37, 2.16) | 1.52 (1.20, 1.93) |  |  |  |
| **Baseline mental illness(es)^c^** | | | |  |  |  |  | 0.001 |  |  |  |  | 0.94 |
| No | | 8584 | 12.7 (1075) | 1 (ref) | 1 (ref) |  | 6082 | 6.5 (415) | 1 (ref) | 1 (ref) |  |  |  |
| Yes | | 873 | 27.5 (224) | 2.17 (1.83, 2.57) | 1.42 (1.14, 1.76) |  | 442 | 9.6 (51) | 1.48 (1.06, 2.08) | 0.99 (0.68, 1.43) |  |  |  |
| **BMI^d^** | | |  |  |  |  | 0.032 |  |  |  |  | 0.40 |  |
| ≤25 | | 5414 | 12.3 (671) | 1 (ref) | 1 (ref) |  | 3858 | 6.4 (267) | 1 (ref) | 1 (ref) |  |  |  |
| >25 | | 4069 | 17.0 (647) | 1.38 (1.20, 1.59) | 1.16 (1.01, 1.34) |  | 2697 | 7.0 (199) | 1.09 (0.87, 1.36) | 1.11 (0.87, 1.42) |  |  |  |
| **Number of acute symptoms^d^** | | |  |  |  |  | <0.001 |  |  |  |  | <0.001 |  |
| 3 or fewer | | 5058 | 5.9 (313) | 1 (ref) | 1 (ref) |  | 3925 | 2.5 (109) | 1 (ref) | 1 (ref) |  |  |  |
| 4-5 | | 2480 | 15.0 (367) | 2.55 (2.10, 3.10) | 2.36 (1.93, 2.88) |  | 1616 | 7.9 (138) | 3.19 (2.35, 4.32) | 3.30 (2.40, 4.54) |  |  |  |
| 6-7 | | 1321 | 27.6 (333) | 4.70 (3.88, 5.69) | 4.38 (3.60, 5.33) |  | 751 | 17.8 (129) | 7.20 (5.33, 9.74) | 7.23 (5.30, 9.87) |  |  |  |
| 8 or more | | 714 | 44.5 (317) | 7.59 (6.33, 9.10) | 6.06 (4.91, 7.48) |  | 321 | 30.3 (98) | 12.29 (9.07, 16.65) | 12.51 (9.01, 17.37) |  |  |  |
| **Hospitalized for covid^d^** | | |  |  |  |  | 0.14 |  |  |  |  | 0.64 |  |
| No | | 9437 | 13.9 (1280) | 1 (ref) | 1 (ref) |  | 6499 | 6.6 (462) | 1 (ref) | 1 (ref) |  |  |  |
| Yes | | 133 | 34.4 (49) | 2.47 (1.80, 3.39) | 1.31 (0.92, 1.87) |  | 111 | 11.3 (12) | 1.71 (0.87, 3.35) | 0.85 (0.43, 1.69) |  |  |  |
| **Other hospitalization** | | |  |  |  |  | 0.84 |  |  |  |  | 0.18 |  |
| No | | 9471 | 14.3 (1312) | 1 (ref) | 1 (ref) |  | 6366 | 6.6 (452) | 1 (ref) | 1 (ref) |  |  |  |
| Yes | | 102 | 17.0 (18) | 1.19 (0.71, 1.99) | 1.06 (0.61, 1.83) |  | 247 | 9.4 (22) | 1.42 (0.85, 2.37) | 1.46 (0.84, 2.54) |  |  |  |
| Abbreviations: BMI, Body Mass Index; CI, Confidence Interval; RR, Relative Risk. | | | | | | | | | | | | | |
| ^a^Percentages, crude and adjusted relative risks, confidence intervals and two-sided *P* values were calculated taking the EpiCov sampling design into account. Percentages were weighted by inverse inclusion probabilities, corrected for non-response and calibrated on the margin of census. Crude and adjusted relative risks were estimated using modified Poisson regressions. Two-sided *P* values were calculated on adjusted models and estimated via Wald tests.  *P* values were calculated on adjusted models and were estimated via Wald tests. | | | | | | | | | | | | | |
| **^b^**Multivariable analysis: variables are mutually adjusted, and adjusted for gender, age, educational level, migratory status, professional status and deprivation status, age of the youngest child in the household, and any history of SARS-Cov-2symptoms among members of the household. | | | | | | | | | | | | | |
| **^c^**Information collected at baseline. | | | | | | | | | | | | | |
| **^d^**Information collected when acute covid symptoms were reported. | | | | | | | | | | | | | |

| **Table S4: Comparison of subjects with observed and missing data on post-covid symptoms regarding auxiliary health-related variables, stratified on the period** | | | | | | | | | | |
| --- | --- | --- | --- | --- | --- | --- | --- | --- | --- | --- |
| *EpiCov French national cohort, 2020-2021* | | | | | | | | |  |  |
|  | | | **Acute covid during the first wave (mid-March to May 2020)** | | | | **Acute covid during the second wave (mid-Sept to Nov 2020)** | | | |
| **Characteristics^a^** | | | **Total** | **Observed data** | **Missing data** | ***P* val^a^** | **Total** | **Observed data** | **Missing data** | ***P* val^*^** |
|  |  |  | **N^a^** | **%(n)^a^** | **%(n)^a^** |  | **N^a^** | **%(n)^a^** | **%(n)^a^** |  |
| **Smoking status^b^** | | |  |  |  | 0.40 |  |  |  | 0.14 |
| Smoker | | 2235 | 92.8 (2080) | 7.2 (155) |  | 1393 | 95.8 (1338) | 4.2 (55) |  |  |
| Non-smoker | | 7950 | 93.5 (7477) | 6.5 (473) |  | 5421 | 96.8 (5261) | 3.2 (160) |  |  |
| **Alcohol consumption^b^** | | |  |  |  | 0.078 |  |  |  | 0.48 |
| Every day | | 971 | 91.3 (898) | 8.7 (73) |  | 578 | 96.5 (560) | 3.5 (18) |  |  |
| ≥ once a week | | 3105 | 94.0 (2932) | 6.0 (173) |  | 2060 | 97.1 (2000) | 2.9 (60) |  |  |
| ≥ once a month | | 1956 | 94.7 (1847) | 5.3 (109) |  | 1302 | 95.9 (1262) | 4.1 ((40) |  |  |
| < once a month | | 1675 | 91.8 (1580) | 8.2 (95) |  | 845 | 97.3 (821) | 2.7 (24) |  |  |
| Never | | 2776 | 93.2 (2598) | 6.8 (178) |  | 2028 | 96.2 (1955) | 3.8 (73) |  |  |
| **Perceived health state^c^** | | |  |  |  | 0.011 |  |  |  | 0.050 |
| Very good | | 2573 | 95.2 (2459) | 4.8 (114) |  | 1853 | 97.7 (1808) | 2.3 (45) |  |  |
| Good | | 5022 | 93.4 (4707) | 6.6 (315) |  | 3345 | 96.7 (3246) | 3.3 (99) |  |  |
| Good enough | | 2195 | 91.5 (2028) | 8.5 (167) |  | 1409 | 95.4 (1346) | 4.6 (63) |  |  |
| Bad or very bad | | 406 | 92.2 (377) | 7.8 (29) |  | 221 | 94.8 (213) | 5.2 (8) |  |  |
| **New condition within the last six months^c^** | | |  |  |  | 0.14 |  |  |  | 0.002 |
| No | | 8961 | 93.6 (8419) | 6.4 (542) |  | 5944 | 96.9 (5775) | 3.1 (169) |  |  |
| Yes | | 1235 | 91.8 (1152) | 8.2 (83) |  | 882 | 94.2 (836) | 5.8 (46) |  |  |
| **New disability within the last six months^c^** | | |  |  |  | 0.34 |  |  |  | 0.083 |
| None | | 7968 | 93.7 (7510) | 6.3 (458) |  | 238 | 97.9 (229) | 2.1 (9) |  |  |
| Mild | | 1843 | 92.5 (1700) | 7.5 (143) |  | 1110 | 95.4 (1063) | 4.6 (47) |  |  |
| Severe | | 385 | 91.8 (360) | 8.2 (25) |  | 5478 | 96.8 (5319) | 3.2 (159) |  |  |
| ^a^ Percentages were weighted by inverse inclusion probabilities, corrected for non-response and calibrated on the margin of census. Numbers are unweighted. Percentages were compared using the second order correction of the Rao and Scott chi-square test. | | | | | | | | | | |
| ^b^Information collected at baseline | | | | | | | | | | |
| ^c^Information collected when post-covid symptoms were reported | | | | | | | | | | |
